# Characterization of the Effect of Increased Plant Density on Canopy Morphology and Stalk Lodging Risk

**DOI:** 10.3389/fpls.2018.01047

**Published:** 2018-09-11

**Authors:** Alam Sher, Aaqil Khan, Umair Ashraf, Hui Hui Liu, Jin Cai Li

**Affiliations:** ^1^School of Agronomy, Anhui Agricultural University, Hefei, China; ^2^Jiangsu Collaborative Innovation Centre for Modern Crop Production, Nanjing, China; ^3^Department of Agronomy, University of Agriculture, Faisalabad, Pakistan

**Keywords:** *Zea mays*, plant density, cultivar, canopy morphology, stalk lodging

## Abstract

Plants react to the environment and to management interventions by undergoing architectural and structural modifications. A field trial was conducted in China in 2016 to study the effects of the plant population on morphological development of the maize canopy. The main objectives of the current study were (i) to characterize the effects of increased plant density on canopy morphology and stalk lodging and (ii) to explore the relationships between organ morphology and stalk lodging. The field experiment was composed of five plant densities (4.5, 6, 7.5, 9, and 15 plants m^−2^) of three cultivars: Zhengdan 958 (lodging-resistant cultivar), Longping 206 and Jinqiu 119 (lodging-susceptible cultivars). In response to plant densities of all the three cultivars, the lamina and sheath lengths increased in lower phytomers but decreased in upper phytomers. The lamina width and internode diameter decreased for all phytomers in response to plant densities for all the cultivars. The correlation between organ morphology, plant density and stalk lodging was linear. Data obtained from characterization used in this study (that is, canopy morphology, correlation of organ morphology with stalk lodging traits in response to various plant densities for different cultivars, etc.) will be useful in future modeling studies to predict the morphology characteristics of the canopy affected by interplant competition and stalk lodging.

## Introduction

With an increase in population, food and energy crises have become the main global challenges. Increased maize production and yield will play a major role toward overcoming these challenges (Cassman et al., 2003; Cassman and Liska, 2007; Grassini et al., 2011). The maize crop provides at least 30% of the caloric requirements of more than 4.5 billion people worldwide; this highlights the importance of maize for ensuring global food security (Von Braun et al., 2010; CIMMYT, 2011). High plant density is one of the main agronomic practices required to achieve maximum yields in modern cropping systems (Yang et al., 2004; Sher et al., 2017; Xu C. et al., 2017; Xu W. et al., 2017; Zheng et al., 2017). However, high plant density may lead to competitive shading within the leaf canopy architecture (Yokozawa and Hara, 1995; Hiyane et al., 2010), thereby limiting interception of radiation by the middle and lower stem leaves particularly during silking time (Tollenaar and Wu, 1999; Maddonni et al., 2001; Christopher et al., 2009; Li and Wang, 2010), accelerating leaf senescence (Tetio-Kagho and Gardner, 1988a; Antonietta et al., 2014), reducing photosynthesis and net assimilation of individual plants. Increasing plant density enhances intra-plant competition, decreases the growth of single-plant crops and accelerates the abortion of young kernels due to limited carbon and nitrogen supply to the ear (Edmeades et al., 2000; Yan et al., 2010). Therefore, understanding the growth response to plant population density (PPD) is of great importance, in order to determine the optimal sowing density, kernel abortion and stalk lodging.

Stalk lodging can be referred to as the breakage of the stalk between the soil level and the main ear insertion node, caused by the complex interaction of several factors (Arnold and Josephson, 1975). Lodging decreases the photosynthetic ability of the plant and biomass production by suppressing the transport of water and nutrients through the xylem and of photosynthetic assimilates via the phloem (Pinthus, 1973; Berry et al., 2004). Moreover, high plant density increases the length of the basal internodes, reduces mechanical tissue thickness and decreases the mechanical cell layers and cortical thickness, while increasing the lodging rate (Huang, 2008; Feng et al., 2010). The tendency of the crop to lodge leads to serious harvesting problems and causes remarkable loss in crop yield, therefore, increasing the demand for grain drying (Berry et al., 2004; Berry and Spink, 2012; Zhang et al., 2014). The annual yield loss is estimated to be 5–40% (Minami and Ujihara, 1991; Nielsen, 2006; Li et al., 2015), and up to 75% of the maize field could be damaged by stalk lodging (Van Dyk, 2001).

The phenotypes of grasses show differences depending on their growth conditions and are affected by abiotic environments (Maddonni and Otegui, 1996; Dingkuhn et al., 2006; Manschadi et al., 2006; Song et al., 2015) and ontogenic contingency (Watson et al., 1995). Architectural plasticity has been widely discussed (Schlichting, 1986; Sultan, 2000; Niklas, 2003; Vandenbussche et al., 2005), and as formulated by de Kroon et al. (2005), can be expressed at the level of individual plant subunits, the whole plant reaction being an integrative by-product. Understanding these responses and making suitable mathematical formalizations and equations is an essential step of crop model development and calibration. Models are useful tools for testing hypotheses on the mechanisms of plant growth and for assessing the potential of crop production and so many ecophysiological models are used for the prediction of crop growth (Carberry et al., 1989; Katawatin et al., 1996; Keating et al., 2003).

The present study specifically focuses on the link between internode morphology and stem lodging. The quantification of this link represents a set of parameters that could be used to expand existing three-dimensional maize models such as ADEL-maize. Therefore, our study attempts to quantify the effects of plant density on maize canopy development and includes cultivar-specific parameters such as organ characteristics under interplant competition. Moreover, the objectives of this paper are to (i) evaluate and characterize the effects of plant density on canopy morphology and stalk lodging for different cultivars and (ii) derive equations that may be used in a functional-structural plant model of maize (e.g., ADEL-maize) (Fournier and Andrieu, 1999) accounting for interplant competition and lodging.

## Materials and Methods

### Site Description

The field experiment was conducted at the Mengchen experimental station in the Huaibei plain in north China (Latitude 33° 944N, Longitude 116° 32, 56 E). Lime concretion black soil in the top 0–20 cm of the arable soil layer, composed of 12.46 g/kg organic matter, 136.60 mg/kg total nitrogen, 17.40 mg/kg available phosphorus and 107 mg/kg available potassium, was used for the experiment. After testing the soil a few days before sowing to determine the nutritional requirement, the basic nutrients applied were 60 kg ha^−1^ of N, 140 kg ha^−1^ of P_2_O_5_, 125 kg ha^−1^ of K_2_O and 15 kg ha^−1^ of ZnSO_4_.7H_2_O, and the soil was initially irrigated with 900 cm^3^ ha^−1^ before crop sowing. The previous crop in the experimental field was wheat.

### Cultural Details and Experimental Design

A completely randomized block design with a split-plot arrangement of five plant densities (subplot treatments), that is, 4.5 plants m^−2^, 6 plants m^−2^, 7.5 plants m^−2^, 9 plants m^−2^ and 15 plants m^−2^ (thereafter referred to as PD4.5, PD6, PD7.5, PD9, and PD15, respectively) was used and each plant density had three replicates. Three maize hybrids (main plot treatment), that is, Zhengdan 958 (ZD958), Longping 206 (LP206), and Jinqiu 119 (JQ119), were manually planted in each row with the help of a sowing drill on June 12, 2016 and harvested on October 5, 2016. The seedlings were thinned during the third leaf stage in each subplot to get the target plant densities. The lowest plant density (4.5 plants m^−2^) was used as the control. Each subplot was 8 m long and 6 m wide and the rows were 60 cm apart in all treatments and in all the replicates. The subplots were separated by a 1 m bare space and the independent replicates were separated by a 1.5 m bare space.

### Sampling and Data Collection

The data were collected according to Song et al. (2015, 2016) and Sher et al. (2016) as discussed below. From each subplot, 10 representative plants were chosen when the plants had five visible leaves (three fully expanded) to guide distinctive samplings of canopy development. The plants were dissected as individual organs for measurement. The rank of each phytomer in the plant was counted acropetally. The total and fully expanded leaf number, lamina length, width, sheath length, internode length and diameter for each phytomer were measured during each destructive sampling. At low plant density, modern maize hybrids rarely produce tillers (Bos et al., 2000). Minimum number of tillers were observed in all plant densities and the analyses of plant data were based on the sampled plants without any tillers. After emergence, a base temperature of 8°C related to the thermal time was used to determine the plant organ development (Song et al., 2015, 2016; Sher et al., 2016).

Lodging was observed under field conditions at the anthesis stage and the lodging percentage was calculated by counting the number of lodged plants to the total number of plants in the whole plot. At the mid-grain filling stage, five plants were harvested from the center of the middle three rows and lodging parameters, that is, stem crushing strength and stem bending resistance, were measured at a middle point of the third basal internode using a stalk strength tester (YYD-1, Top Instrument Co., Ltd, Zhejiang, China). For these measurements, third basal internodes with the stem leaf and sheath were removed and were placed on the supporting pillars at a distance of 5 cm each. The tester was set perpendicularly to the middle of the internode, which lodged gradually, and the stem bending resistance (N) and crushing strength (N) were measured when the basal internode was pushed to its breaking point. Stem bending resistance and crushing strength were expressed in Newtons (N). Stem bending resistance was also measured at 15° at the third basal internode, with the help of a protractor.

### Data Analysis

Statistical analyses at a significance level of *p* = 0.05 on individual phytomers were carried out using *t*-test (MS Excel, Microsoft Inc., 121 Seattle, WA, United States). Only two treatments, that is, PD9 and PD15 with the largest difference were chosen for a clearer representation of the effect of interplant competition on organ development. The difference (%) was then calculated using the below formula if the difference between the treatments was significant.

(1) (PD15−PD45)/PD4.5*100%

The correlations between morphological parameters and lodging parameters, according to various plant densities, (that is, basal third internode diameter and internode diameter/length), were quantified with a linear regression function (*y = ax + b*).

## Results

### Characterization of the Final Lamina Length Response to Increased Plant Density

**FIGURE 1:**
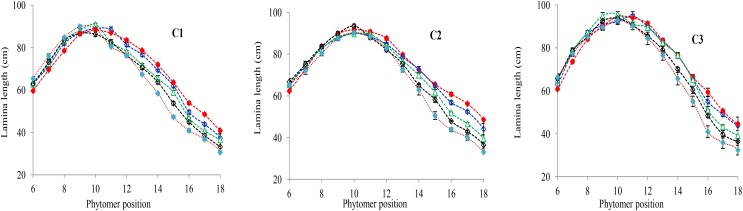
Final lamina length differences between plant densities [PD4.5 (red dashed line), PD6 (blue dashed line), PD7.5, (green dashed line), PD9 (black dashed line), and PD15 m^−2^ (pink dashed line)] and cultivars (C1 Zhengdan 958, C2 Longping 206, and C3 Jinqiu 119). Vertical bars indicate standard errors.

The final lamina lengths for lower and upper phytomers significantly increased and decreased with increase in plant density. The final lamina lengths at different phytomer positions among plant densities across various cultivars are represented in **Figure 1**. When the plant density increased from PD4.5 to PD6 and PD7.5 all the phytomers responded positively. However, none of the phytomers responded when the plant density increased from PD7.5 to PD9 and PD15, for all cultivars, due to mild competition. For lower phytomers the lamina lengths increased at greater plant densities for all cultivars, and there was even more increase at ranks 7, 8, and 9. For upper phytomers, the lamina lengths decreased for all plant densities, but at greater plant densities the decrease was more as compared with other densities due to more interplant competition. The differences of final lamina lengths between plant densities for cultivars with phytomer positions were quantified by a linear regression function, as for phytomers 11–18 for all cultivars, and are presented in **Table 1**.

**Table 1:** Equations of the final lamina length (cm) response to increased plant densities for different cultivars.

PP		11	12	13	14	15	16	17	18
**C1**	*a*	−0.70	−0.64	−1.04	−1.25	−1.60	−1.13	−0.99	−0.90
	*b*	90.09	84.60	81.83	76.40	70.64	56.79	50.12	43.29
	*R*^2^	0.69	0.65	0.85	0.94	0.95	0.88	0.77	0.89
**C2**	*a*	−0.16	−0.38	−0.67	−1.01	−1.49	−1.56	−1.47	−1.38
	*b*	90.32	87.43	82.22	77.06	72.69	65.28	59.98	51.85
	*R*^2^	0.78	0.89	0.95	0.85	0.98	0.86	0.80	0.83
**C3**	*a*	−0.50	−0.67	−0.71	−1.14	−1.14	−1.68	−1.42	−1.20
	*b*	96.04	93.75	86.87	82.62	72.10	65.01	55.60	49.39
	*R^2^*	0.72	0.82	0.92	0.87	0.95	0.95	0.87	0.90

*Here C1 Zhengdan 958, C2 Longping 206, C3 Jinqiu 119, PP ranks. R^2^ is statistically significant (P < 0.05).*

### Characterization of the Final Lamina Width Response to Increased Plant Density

**FIGURE 2:**
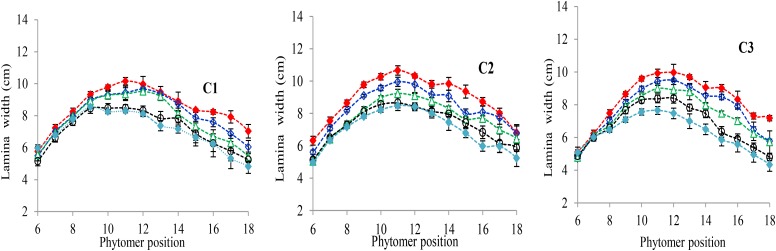
Final lamina width differences between plant densities [PD4.5 (red dashed line), PD6 (blue dashed line), PD7.5, (green dashed line), PD9 (black dashed line), and PD15 m^−2^ (pink dashed line)] and cultivars (C1 Zhengdan 958, C2 Longping 206, and C3 Jinqiu 119). Vertical bars indicate standard errors.

The final lamina widths significantly decreased for all phytomers in all hybrids in response to plant densities (**Figure 2**). At plant densities of PD4.5, PD6 and PD7.5, the decrease was less as compared with densities of PD9 and PD15. The differences of the final lamina widths between plant densities for cultivars with upper phytomer positions were quantified by a linear regression function as for phytomers 11–18 for C2 and 10–18 for C1 and C3, respectively, because these phytomers showed a higher response compared with the other phytomers (**Table 2**).

**Table 2:** Equations of the final lamina width (cm) response to increased plant densities for different cultivars.

PP		10	11	12	13	14	15	16	17	18
**C1**	*a*	−0.12	−0.15	−0.17	−0.18	−0.12	−0.14	−0.18	−0.23	−0.18
	*b*	10.12	10.48	10.56	10.24	9.2102	8.6364	8.5123	8.3687	7.1836
	*R*^2^	0.76	0.74	0.75	0.73	0.73	0.72	0.68	0.81	0.72
**C2**	*a*	–	−0.191	−0.172	−0.157	−0.208	−0.225	−0.254	−0.197	−0.152
	*b*	–	11.009	10.658	10.105	10.271	9.8834	9.5964	8.6302	7.4411
	*R*^2^		0.73	0.69	0.74	0.80	0.85	0.93	0.78	0.97
**C3**	*a*	−0.179	−0.211	−0.233	−0.252	−0.251	−0.299	−0.264	−0.210	−0.238
	*b*	10.089	10.64	10.8	10.595	9.7349	9.9759	9.1595	7.8331	7.526
	*R^2^*	0.91	0.93	0.95	0.92	0.60	0.85	0.80	0.83	0.76

*Here C1 Zhengdan 958, C2 Longping 206, C3 Jinqiu 119, PP ranks. R^2^ is statistically significant (P < 0.05).*

### Characterization of the Final Sheath Length Response to Increased Plant Density

**FIGURE 3:**
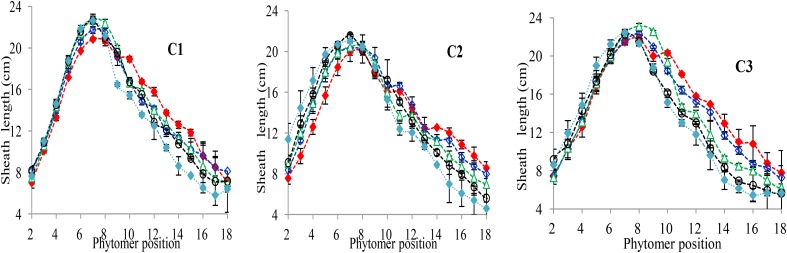
Final sheath length differences between plant densities [PD4.5 (red dashed line), PD6 (blue dashed line), PD7.5, (green dashed line), PD9 (black dashed line), and PD15 m^−2^ (pink dashed line)] and cultivars (C1 Zhengdan 958, C2 Longping 206, and C3 Jinqiu 119). Vertical bars indicate standard errors.

The final sheath lengths significantly increased for lower phytomers and significantly decreased for upper phytomers for all cultivars, and the differences of the final sheath lengths in response to various plant densities with phytomer positions are represented in **Figure 3**, which shows an increase for lower phytomers and a decrease for upper phytomers; the increase and decrease were less at PD4.5, PD6, and PD7.5 compared with at PD9 and PD15. The differences of final sheath lengths between plant densities for cultivars with upper phytomer positions were quantified by a linear regression function for phytomers 10–18 for C1 and C3, and for phytomers 11–18, for C2 because these phytomers responded more compared with other phytomers (**Table 3**).

**Table 3:** Equations of the final sheath length (cm) response to increased plant densities for different cultivars.

PP		10	11	12	13	14	15	16	17	18
**C1**	a	−0.26	−0.25	−0.27	−0.27	−0.35	0.36	−0.31	−0.27	−0.27
	b	19.07	17.44	16.15	14.50	13.89	12.87	11.05	9.75	9.25
	R^2^	0.69	0.73	0.73	0.82	0.97	0.92	0.94	0.95	0.94
**C2**	a	–	−0.53	−0.25	−0.18	−0.30	−0.47	−0.43	−0.40	−0.38
	b	–	18.35	15.75	13.31	13.31	13.75	12.26	11.00	9.95
	R^2^	–	0.78	0.90	0.92	0.94	0.93	0.95	0.90	0.89
**C3**	a	−0.48	−0.44	−0.38	−0.51	−0.54	−0.46	−0.43	−0.30	−0.19
	b	21.88	18.97	17.16	16.40	14.41	12.39	11.55	9.63	8.15
	*R^2^*	0.78	0.79	0.90	0.77	0.82	0.81	0.77	0.75	0.89

*Here C1 Zhengdan 958, C2 Longping 206, C3 Jinqiu 119, PP ranks. R^2^ is statistically significant (P < 0.05).*

### Characterization of the Final Internode Length Response to Increased Plant Density

**FIGURE 4:**
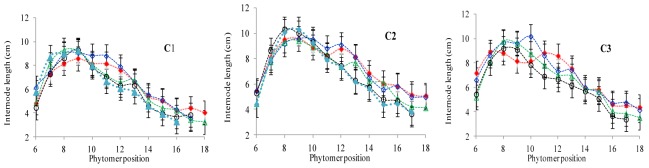
Final internode length differences between plant densities [PD4.5 (red dashed line), PD6 (blue dashed line), PD7.5, (green dashed line), PD9 (black dashed line), and PD15 m^−2^ (pink dashed line)] and cultivars that is (C1 Zhengdan 958, C2 Longping 206, and C3 Jinqiu 119). Vertical bars indicate standard errors.

The final internode lengths at different phytomer positions in response to plant densities across various cultivars are represented in **Figure 4**. The differences in final internode lengths among plant densities for cultivars with phytomer positions were quantified by a linear regression function for phytomers 11–17 for all cultivars, because these phytomers responded more as compared with other phytomers, and the lengths of lower phytomers with plant density were not consistent (**Table 4**). Lower internodes were found to be more sensitive to increased interplant competition than upper internodes, across all cultivars.

**Table 4:** Equations of the final internode length (cm) response to increased plant densities for different cultivars.

PP		11	12	13	14	15	16	17
**C1**	*a*	−0.17	−0.16	−0.09	−0.09	−0.12	−0.10	−0.10
	*b*	8.99	8.27	7.19	5.77	5.44	4.81	4.50
	*R^2^*	0.62	0.66	0.86	0.67	0.69	0.88	0.62
**C2**	*a*	−0.10	−0.15	−0.21	−0.11	−0.16	−0.14	−0.14
	*b*	8.77	9.25	9.01	7.15	6.74	6.27	5.49
	*R*^2^	0.62	0.69	0.74	0.78	0.75	0.82	0.79
**C3**	*a*	−0.25	−0.15	−0.16	−0.05	−0.15	−0.04	−0.08
	*b*	9.78	8.50	8.17	6.23	6.55	4.56	4.70
	*R*^2^	0.87	0.85	0.82	0.94	0.95	0.86	0.82

*Here C1 Zhengdan 958, C2 Longping 206, C3 Jinqiu 119, PP ranks. R^2^ is significant (P < 0.05).*

### Characterization of Final Internode Diameter Response to Increased Plant Density

**FIGURE 5:**
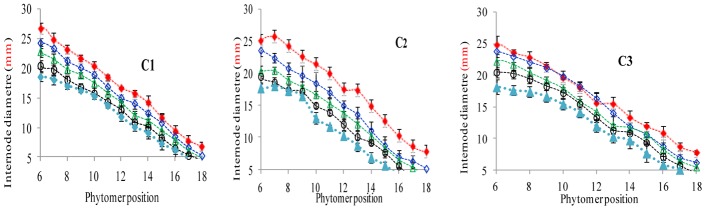
Final internode diameter differences between plant densities [PD4.5 (red dashed line), PD6 (blue dashed line), PD7.5, (green dashed line), PD9 (black dashed line) and PD15 m^−2^ (pink dashed line)] and cultivars (C1 Zhengdan 958, C2 Longping 206, and C3 Jinqiu 119). Vertical bars indicate standard errors.

The final internode diameters were significantly reduced for all cultivars. The differences between plant densities and cultivars for internode diameter are represented in **Figure 5**, which shows a decrease for all phytomers; the decrease was less in PD4.5, PD6, and PD7.5 as compared with PD9 and PD15. The characterization of the final internode diameters between plant densities with phytomer positions was quantified by a linear regression function for phytomers 7–18 for all cultivars, because these phytomers responded more as compared with other phytomers (**Table 5**).

**Table 5:** Equations of the final internode diameter (mm) response to increased plant densities for different cultivars.

PP		7	8	9	10	11	12	13	14	15	16	17	18
**C1**	*a*	−0.56	−0.50	−0.46	−0.42	−0.43	−0.50	−0.44	−0.41	−0.29	−0.26	−0.21	−0.14
	*b*	24.59	22.97	21.47	19.45	17.78	16.83	15.08	12.93	10.18	8.36	6.89	5.69
	*R*^2^	0.82	0.77	0.76	0.78	0.85	0.76	0.80	0.85	0.74	0.79	0.66	0.72
**C2**	*a*	−0.56	−0.50	−0.73	−0.72	−0.64	−0.73	−0.66	−0.54	−0.49	−0.50	−0.47	−0.42
	*b*	24.48	22.93	23.07	21.61	19.11	18.48	15.94	13.12	10.78	9.50	8.20	7.31
	*R*^2^	0.62	0.66	0.83	0.85	0.85	0.76	0.81	0.76	0.75	0.79	0.70	0.69
**C3**	*a*	−0.52	−0.48	−0.43	−0.40	−0.41	−0.46	−0.32	−0.39	−0.43	−0.30	−0.38	−0.30
	*b*	24.81	23.36	21.66	19.71	17.69	16.53	14.13	13.30	11.73	9.07	8.63	7.00
	*R*^2^	0.93	0.90	0.91	0.82	0.84	0.79	0.88	0.95	0.85	0.76	0.83	0.69

*Here C1 Zhengdan958, C2 Longping 206, C3 Jinqiu 119, PP ranks. R^2^ is significant (P < 0.05).*

### Relationships and Correlation Between Stalk Lodging Parameters and Plant Density Level

**FIGURE 6:**
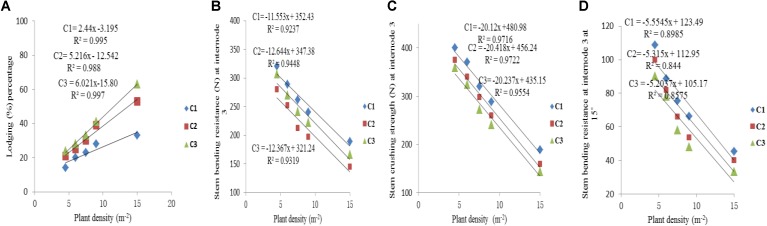
Stem lodging characteristics, that is lodging percentage **(A)**, stem bending resistance at internode 3 **(B)**, stem crushing strength at internode 3 **(C)**, stem bending resistance at internode 3 at 15° **(D)** between plant densities PD4.5, PD6, PD7.5, PD9, PD15 m^−2^, and cultivars (C1 Zhengdan 958, C2 Longping 206, C3 Jinqiu 119).

The final internode lengths were increased for lower phytomers and decreased for higher phytomers for all densities, whereas the final internode diameters were decreased for all phytomers with planting densities for all cultivars. The data on stem lodging characteristics that is lodging percentage, stem bending resistance and stem crushing strength at internode 3 and stem bending resistance at 15° at internode 3, are presented in **Figure 6** with a linear regression function. The overall lodging percentage was less in PD4.5, PD6, and PD7.5 as compared with PD9 and PD15 due to thick and compact internodes. At PD4.5 and PD6, the internode diameter was thicker, stronger and more compact because the stem bending resistance, stem crushing strength and other lodging characteristics, like stem bending at 15°, were significantly greater relative to PD7.5. At PD9, the internode length was increased whereas the internode diameter was decreased, due to severe competition, which reduced stem bending resistance, stem crushing strength and other lodging characteristics and resulted in more stem lodging. At PD15 there was no further decrease, but the growth stopped due to severe competition. Among the cultivars, ZD958 had the thickest and the most compact internodes, whereas the internode length and lodging percentage (%) were less; this could be explained by the fact that this cultivar is considered to be lodging-resistant as compared with LP206 and JQ119. Stem lodging characteristics data are presented in **Table 6**.

**Table 6:** Equations of the lodging parameters: stem bending resistance (N), stem crushing strength (N) at basal internode three.

PP		Lodging % at anthesis stage	SBR (N) at internode three	SCS (N) at internode three	SBR (N) at internode three at 15°
**C1**	*a*	2.444	−11.553	−20.12	−5.5545
	*b*	−3.195	352.43	480.98	123.49
	*R*^2^	0.995^∗∗^	0.9237^∗∗^	0.9716^∗∗^	0.8985^∗∗^
**C2**	*a*	5.216	−12.644	−20.418	−5.315
	*b*	− 12.542	347.38	456.24	112.95
	*R*^2^	0.988^∗∗^	0.9448^∗∗^	0.9722^∗∗^	0.844^∗∗^
**C3**	*a*	6.021	−12.367	−20.237	−5.2037
	*b*	− 15.80	321.24	435.15	105.17
	*R*^2^	0.997^∗∗^	0.9319^∗∗^	0.9554^∗∗^	0.8575^∗∗^

*Where SBR is stem bending resistance (N), SCS is stem crushing strength (N), C1 is Zhengdan 958, C2 is Longping 206, C3 is Jinqiu 119. R^2^ is significant ^∗∗^(p < 0.01), ^∗^(p < 0.05).*

### The Relationship Between Lodging Parameters and Internode Morphology (Diameter, Length, and Diameter/Length)

**FIGURE 7:**
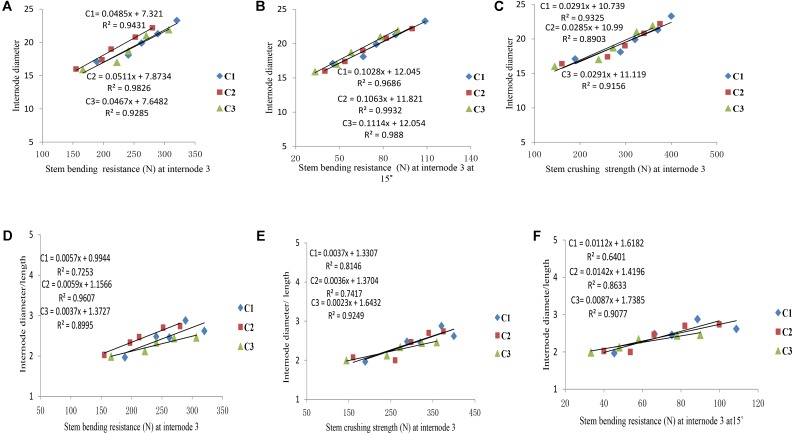
Correlation of internode morphology with stalk lodging characteristics, that is, **(A)** internode diameter (mm) with stem bending resistance at internode three, **(B)** internode diameter with stem crushing strength at internode three, **(C)** internode diameter with stem bending resistance at internode three at 15°, **(D)** internode diameter/length with stem bending resistance at internode three, **(E)** internode diameter/length with stem crushing strength at internode three, **(F)** internode diameter/length with stem bending resistance at internode three at 15° between plant densities (PD4.5, PD6, PD7.5, PD9, and PD15 m^−2^) and cultivars (C1 Zhengdan 958, C2 Longping 206, and C3 Jinqiu 119).

Final internode diameters were decreased for all phytomers with planting densities for all cultivars, but the final internode lengths were not consistent with all densities. The correlations of lodging parameters like stem bending resistance (N) and stem crushing strength (N) at internode 3 with internode morphology, that is, internode diameter and internode diameter/length, are presented in **Figure 7**. Internode morphology, that is internode diameter and internode diameter to length ratio, is linearly correlated with lodging parameters; as internode diameters increased stem bending resistances and stem crushing resistances also increased. Among cultivars, the internode morphology correlation of ZD958 with lodging parameters was best as compared with LP206 and JQ119. Correlations of stem lodging characteristics data with internode morphology quantified with linear regression function parameters are presented in (**Table 7**).

**Table 7:** Equations of the lodging parameters: stem bending resistance (N), stem crushing strength (N) at basal internode three with internode morphology.

		Correlation of SBR and ID at internode three	Correlation of SCS and ID at internode three	Correlation of SBR and ID at internode three at 15°	Correlation of SBR and ID/L at internode three	Correlation of SCS ID/L at internode three	Correlation of SBR and ID/L at internode at three 15°
**C1**	*a*	0.0485	0.1028	0.0291	0.0057	0.0037	0.0112
	*b*	7.321	12.045	10.739	0.9944	1.3307	1.6182
	*R*^2^	0.9431^∗∗^	0.9686^∗∗^	0.9325^∗∗^	0.7253^∗∗^	0.8146^∗∗^	0.7834^∗^
**C2**	*a*	0.0511	0.1063	0.0285	0.0059	0.0036	0.0142
	*b*	7.8734	11.821	10.99	1.1566	1.3704	1.4196
	*R*^2^	0.9826^∗∗^	0.9932^∗∗^	0.8903^∗∗^	0.9607^∗∗^	0.7417^∗∗^	0.8633^∗∗^
**C3**	*a*	0.0467	0.1114	0.0291	0.0037	0.0023	0.0087
	*b*	7.6482	12.054	11.119	1.3727	1.6432	1.7385
	*R^2^*	0.9285^∗∗^	0.988^∗∗^	0.9156^∗∗^	0.8995^∗^	0.9249^∗∗^	0.9077^∗^

*Here SBR is stem bending resistance, SCS is stem crushing strength, ID is internode diameter, ID/L is internode diameter/length and C1 Zhengdan 958, C2 Longping 206, C3 Jinqiu 119. R^2^ is significant ^∗∗^(p < 0.01), ^∗^(p < 0.05).*

## Discussion

### Morphological Characteristics in Response to Various Planting Densities and Cultivars

The plant and canopy architectures determine the optimum plant density, mainly influencing photosynthetic efficiency, disease resistance and lodging resistance. At the same time, plant morphology is a consequence of light interception and partitioning. Light interception by the canopy is greatly influenced by the leaf angle (Lee and Tollenaar, 2007; Hammer et al., 2009). Unlike previous studies which only considered one hybrid and either four plant density levels (2, 6, 12, and 20 plants m^−2^, Song et al., 2016) or two levels (9.5 and 30.5 plants m^−2^_,_ Andrieu et al., 2006), the present study looked at three cultivars (ZD958, LP206 and JQ119) at five densities (4.5, 6, 7.5, 9, and 15 plants m^−2^). This study was conducted to investigate organ development and stalk lodging in response to various plant densities from low to high interplant competition, identifying different response characteristics to high interplant competition with three cultivars. The effect of plant density on lamina length and sheath length showed two different patterns, that is, increase for the lower phytomers and decrease for the upper phytomers for all cultivars (**Figures 1**, **3**), but the increase and decrease in PD4.5 and P6 were more as compared with PD7.5, while the growth was suppressed at PD9 and PD15 due to severe competition; this is similar to the findings of others (Andrieu et al., 2006; Song et al., 2016). The decrease in the lamina and the sheath lengths in the upper ranks may be due to the low carbohydrate availability in response to high interplant competition. The lamina width and internode diameter were reduced for all cultivars (**Figures 2**, **5**) for lower and upper phytomers by interplant competition, and the effect took place as soon as PD4.5 was suppressed and even remained after PD9, confirming results from previous studies (Sher et al., 2016; Song et al., 2016). The internode length (**Figure 4**) for lower ranks was greater at high plant densities, PD9 and PD15, as compared with PD4.5, PD6, and PD7.5 for all cultivars. Plants grown under a crowded canopy received different quality of light, enriched with far-red (FR) and impoverished in red (R) radiations (Tetio-Kagho and Gardner, 1988b: Sattin et al., 1994; Xue et al., 2016). This high FR/R ratio triggered many morphological changes in plant architecture, stimulating internode elongation, favoring apical dominance and decreasing internode diameter (Troyer and Rosenbrook, 1991; Rajcan and Swanton, 2001). The extent of the internode elongation promotion in response to high plant density reduced as the number of phytomers increased. The difference may be due to the bottom internodes receiving less radiation as the canopy depth increased. This caused an increase in the internode length and decrease in the internode diameter, leading them to become slimmer and making them more prone to lodging (Song et al., 2016). Our results are similar to those of Xiang et al. (2016), who showed that as plant density in wheat increased, the length of the first internode also increased. The response seen in JQ119 was more with respect to lamina length and width for all phytomers as compared with ZD958 and LP206. The measured decrease in internode diameter was greater in JQ119 for all phytomers as compared with ZD958 and LP206. Our results are similar to those of Shi et al. (2016) who used two plant densities and two cultivars, ZD958 and LD981, and found that morphological parameters like stem diameter and stem thickness reduced with an increase in plant density.

### Lodging Risk in Response to Increased Plant Density and Cultivars

As the plant density increases the internodes become thinner, making the plant more prone to stalk lodging (Song et al., 2016). The stalk lodging risk at PD4.5, PD6, and PD7.5 due to compact and thick internodes was less as compared with PD9 and PD15 due to less interplant competition. In our study, 60% more lodging was observed at high plant density at basal internode three as compared with other densities (**Figure 6A**) which is similar to the reports by Gou et al. (2008, 2010). Compared with the flowering and anthesis stages, the translocation of carbohydrates to grains becomes higher at the grain filling stage to fulfill the seed requirement. The stored carbohydrates in the maize stalks were transported to grains and weakened the basal internodes, thus reducing the bending quality and providing an ease of lodging (Xue et al., 2016); this is because the basal internodes act as a lever for holding the plants upright (Yuan et al., 2002). The stem bending resistance and crushing resistance were also less at PD4.5, PD6, and PD7.5 as compared with PD9 and PD15 (**Figure 6**). Morphological characteristics that correlated with stalk lodging were the plant height, the diameter and the length of the basal internode, the thickness of the rind and the weight of the 5 cm basal section (Esechie, 1985). Light intensity and quality are also the main sources of photosynthetically active radiation which directly govern photosynthesis. Decline in PAR in close planting due to more interplant competition causes a decrease in the photosynthetic capacity of leaves in the lower canopy (Li et al., 2014; Marchiori et al., 2014). Preliminary research reported that leaves below the cob are the main source of assimilation of photosynthates for the growth of maize stalk (Maddonni and Otegui, 2004). Therefore, a decline in PAR in the lower canopy may result in less accumulation of dry matter in the basal internode, decreasing the thickness of the mechanical tissue in the stalk (Kaack et al., 2003; Yao et al., 2013), which may lead to a decrease in stem bending and crushing strength of basal internode three at high plant density. Our results are similar to those of previous research, which showed that there was a significant decrease in the stalk crushing strength, mechanical tissue thickness and internode diameter whereas the length of the basal internode and lodging rate increased (Gou et al., 2007; Feng et al., 2014; Xiang et al., 2016). Our results are inconsistent with those of Kuai et al. (2015, 2016) which states that lodging resistance increased with high plant density. While comparing ZD958, JQ119, and LP206, the decrease in the internode diameter was more at PD9 and PD15 as compared with PD4.5, PD6, and PD7.5. ZD958 is a better lodging resistant cultivar with compact, shorter, thicker and stronger internodes. ZD958 also performed better for stalk lodging characteristics (like lodging percentage, stem bending resistance and stem crushing strength) and correlation of stalk lodging characteristics with internode morphology at basal internode three as compared with LP206 and JQ119. Our results are similar to those of Yokozawa and Hara (1995); Wang and Frei (2011), and Shi et al. (2016), who reported that maize plants become taller when plant density increased as the mutual shading and the lodging percentage increased, although there is considerable varietal variation in this characteristic. The relationship and correlation between stalk lodging characteristics with internode morphology, that is, internode diameter, internode diameter/length (**Figure 7**), were linear.

### Characterization for Increasing Plant Density on Maize Morphology and Lodging Risk

The canopy morphology in response to plant density is considered to be one of the most important agronomic traits. It has since long attracted the attention of breeders, as the prediction of morphology (in an effort to achieve the ideal plant architecture) could be a tool for improving the grain yield. Maize organ allometry is an approach that represents the robust linkage between the morphology and the physiological processes at the organ level, enabling crop models to represent crop growth and development more faithfully to the underlying biological processes (Song et al., 2015). In a previous study (Sher et al., 2016), the effect of plant density on canopy morphology was quantified using linear and polynomial regression equations between two consecutive plant densities. This study characterized the effect of increased plant density on canopy morphology and explored the relationship between organ morphology and stalk lodging with different cultivars. The characterization of the lamina length between plant densities for cultivars with phytomer position was fitted by a linear regression function (**Table 1**) for the upper phytomers because these phytomers responded more when the value of coefficient *b* was reduced and the decrease in JQ119 was more compared with LP 206 and ZD958 for the upper phytomers. The sharp reductions in lamina width among plant densities and cultivars in relation to phytomer position were fitted with linear functions (**Table 2**), suggesting that the growth of leaf width was more prone to interplant competition as compared with the growth of leaf length; the leaf shape changed due to high interplant competition (narrow leaves). The sheath length and the internode length were fitted with a linear regression equation (**Tables 3**, **4**) for the upper phytomers because these phytomers responded more for all the cultivars. The internode diameter was also fitted with a linear regression function (**Table 5**), showing a sharp reduction from the upper to the lower positions for all the phytomers due to smaller growth rate because of the severe competition and low light intensity for all cultivars. The characterization of stalk lodging characteristics with plant density (**Table 6**) and with internode morphology, that is, internode diameter, internode diameter/length (**Table 7**), was linear and can be used for future modeling studies. Consequently, the organ length response to plant density could be due to the light signal and the interplant competition but independent of assimilate availability (Maddonni et al., 2002; Song et al., 2016). It should be noted that a density of 4.5 plants m^−2^ is generally not a practical PPD in maize production. A plant density of around 6 plants m^−2^ or 7.5 plants m^−2^ is commonly used.

## Conclusion

This study compared canopy development in response to various plant densities and corn cultivars. The extension of both the lamina and the sheath was promoted in the lower phytomers and decreased in the upper phytomers in all hybrids. The lamina width and internode diameter were reduced in all the phytomers. Less competition was observed between different organs at lower plant densities wherein the greatest competition was observed at PD15. The relationship between organ development and plant density was quantified. Linear regression functions with different phytomer positions as input were identified when quantifying the changes in internode morphology between different plant densities and cultivars. These functions can be used to predict the effect of interplant competition on internode morphology and stalk lodging. Due to the compact and thick internode morphology, and consequently better lodging resistance, ZD958 showed better morphological characteristics than LP206 and JQ 119. Moreover, the findings of this study will be used for predicting the morphological characteristics of the canopy affected by different plant densities and stalk lodging risks.

## Author Contributions

JL and HL conceived and designed the research. AS and AK performed the research and wrote the manuscript. UA analyzed the data and helped in interpretation of the result and critically revised the final version of manuscript.

## Conflict of Interest Statement

The authors declare that the research was conducted in the absence of any commercial or financial relationships that could be construed as a potential conflict of interest.
